# A Semantic Autonomous Video Surveillance System for Dense Camera Networks in Smart Cities

**DOI:** 10.3390/s120810407

**Published:** 2012-08-02

**Authors:** Lorena Calavia, Carlos Baladrón, Javier M. Aguiar, Belén Carro, Antonio Sánchez-Esguevillas

**Affiliations:** Universidad de Valladolid, Dpto. TSyCeIT, ETSIT, Paseo de Belén 15, Valladolid 47011, Spain; E-Mails: cbalzor@ribera.tel.uva.es (C.B.); javagu@tel.uva.es (J.M.A.); belcar@tel.uva.es (B.C.); antsan@tel.uva.es (A.S.-E.)

**Keywords:** smart sensors, surveillance, semantics, safety and security

## Abstract

This paper presents a proposal of an intelligent video surveillance system able to detect and identify abnormal and alarming situations by analyzing object movement. The system is designed to minimize video processing and transmission, thus allowing a large number of cameras to be deployed on the system, and therefore making it suitable for its usage as an integrated safety and security solution in Smart Cities. Alarm detection is performed on the basis of parameters of the moving objects and their trajectories, and is performed using semantic reasoning and ontologies. This means that the system employs a high-level conceptual language easy to understand for human operators, capable of raising enriched alarms with descriptions of what is happening on the image, and to automate reactions to them such as alerting the appropriate emergency services using the Smart City safety network.

## 1. Introduction

Advances in Information and Communication Technologies (ICTs) are triggering a transformation of the environments where we live into intelligent entities globally known as Smart Spaces (Smart Homes, Smart Buildings, Smart Cities, *etc.*). They capture information using large sensors networks distributed throughout its domain (a house, a building, a whole city, *etc.*) and use it to intelligently adapt their behaviour to the needs of the users [1].

Additionally, modern society is experiencing an increasing interest in safety and security, resulting in many experiences related to wide area deployment of video surveillance systems. When integrated within Smart Spaces, these video sensor networks confer to the intelligent system the ability to watch the environment, and when combined with their intelligence, to detect and identify different abnormal situations that may arise inside the Smart domain. Currently there is a wide range of video surveillance systems that are used in different fields such as intrusion detection or traffic surveillance [2–4]. However, autonomous detection of alerts and abnormal situations is still at a primitive stage.

Automatic object recognition is therefore a hot topic with quite a lot of literature behind it, such as [5–8]. When the system is capable of identifying objects, artificial intelligence (AI) and video interpretation algorithms are capable of detecting abnormal behaviours of those objects, mainly using two different strategies: statistical and semantic analysis.

Usage of statistical analysis to process visual information is discussed for instance in [9,10]. Focused on video surveillance and the usage of “Latent Semantic Analysis”, the authors present probabilistic models where statistical classification and relational learning are applied to identify recurrent routines.

The literature reports some systems hardcoded for their operation in predefined and highly controlled locations, such as [7,11] (which perform statistical processing of images in order to recognize and track different objects) or [10,12,13] (aimed at statistical behaviour detection and role assignment to objects). Porting them to real life environments is difficult because their low flexibility: the system has to be completely redesigned and adapted for each domain.

Semantic knowledge representation and processing is a discipline that was introduced in the information technologies landscape about 10 years ago [14]. These semantic technologies have been developed to overcome the limitations of traditional syntactic/statistical data management and representation, and are being applied profusely in the new generation of the World Wide Web, which has sometimes been labeled as Web 3.0 or Semantic Web [15]. However, semantics are also being applied to new application scenarios that can benefit from the structured knowledge representation and reasoning (providing advantages like interoperability between heterogeneous systems, ability to infer relationships that are not explicitly stored in databases, *etc.*) [16,17].

Several sources [9,11,18,19] propose the usage of machine vision algorithms for detecting the presence of a set of fixed objects in a video stream. Once the objects are detected, the characterization of normal and abnormal behaviour by the inclusion of a semantic knowledge model could be achieved. Some authors [20,21] present Semantic Information Fusion, where raw sensor data are converted to semantic data so that the application layer processes the resulting semantic interpretations using a language with high-level abstractions. Some systems classify the different regions of the image [14,22–24] or visual templates [16]; others [20,21] focus on the processing of a low-level image, using Bayesian methods.

This semantic data processing usually comprises two stages: first, knowledge model specification; and second, pattern recognition and matching. The first of these phases is carried out off-line, at design time, and implies the creation of an ontology which describes the domain of knowledge where the system operates in terms of the entities implied and the relationships among them [10,12,13,15,25–27]. This knowledge model is employed in the second phase which performs the semantic interpretation of the input data according to the domain knowledge model specified in the first stage.

However, all of the previous cases involve a high computational load, because the algorithms operate directly over the images. This means that either all cameras have to include high performance processors or the video signal has to be completely sent to the control center where the intelligent algorithms are run. For deployments of dense surveillance networks (which is normally the case of Smart Environments), this is highly inefficient, since cameras have to be very expensive or a huge bandwidth is required.

This work aims at providing a solution to this problem by designing and developing an automated video surveillance system suitable for dense deployments in Smart Spaces, capable of working with small and cheap cameras, small bandwidth and optimizing processing power.

The approach followed by the system proposed in this work is based on a three stage processing scheme: first, detecting objects in motion at the cameras to avoid sending large video data, while at the same time keeping the processing power required by the cameras low avoiding the application of complex, resource intensive, object identification algorithms; second, automatically building at the control center a route model of the moving objects in the watched scenes using the movement parameters identified by the cameras; and third, performing semantic reasoning over the route model and the movement parameters to identify alarms at the conceptual level, that is, not only identifying that an unusual event is happening, but identifying the nature of that event (a car crash, a fire, an intrusion, *etc.*). The work presented along this paper has been carried out within the European project CELTIC HuSIMS (Human Situation Monitoring System).

After this introduction, Section 2 presents overall system design. Section 3 explains the operation of the different stages of the system (cameras, route detection algorithm and semantic reasoning over the processed data). Section 4 presents three real use cases implemented across two different surveillance domains. Section 5 gives the numerical results of the performance and accuracy experiments of the system. Section 6 discusses the features of the proposed solution against other similar options documented in the literature. Finally, Section 7 summarizes the conclusions of this work.

## 2. High Level System Design and Architecture

As described in the introduction, the aim of this work is to design and develop an intelligent/autonomous video interpretation system based on semantic reasoning to be deployed on a massive video surveillance network with thousands of cameras, capable of covering an area similar to a city. More precisely, the objectives of this work are:

1. Develop an algorithm for the interpretation of video scenes and identification of alarm situations.
2. The system should be capable of giving rich, human-level information about the alarm. It would not be enough to say that there has been an alarm, the system should say, for instance, that there has been a car crash.
3. The system should operate on large numbers of cheap cameras to allow a wide area deployment. This means cameras will not have enough processing power to run complex object identification algorithms, and that it is impossible for every camera to send a detailed video signal to the control room for its real-time analysis. This scenario is the one found in “Smart City” deployments, *i.e.*, intelligent urban-scale systems.
4. The system should be able to operate in all the different knowledge domains related to surveillance in the Smart City scenario. For instance, it should be able to handle traffic control, fire alarms, crowd control and vandalism detection.

Current state-of-the-art surveillance systems are based either on statistical analysis of image features or on the hard-coded interpretation of object identification. Statistical alarm detection simply identifies abnormal behaviours, understanding abnormal as things that do not happen frequently according to a certain mathematical criteria, so it is impossible for them to fulfill requirement number 2. Systems based on hard-coded interpretations work on the basis of a hardcoded rule engine and do not usually make use of formal semantic technologies. This means that it is necessary to manually modify the specific implementation of the algorithms in the system to port it from one domain to the other, if that is feasible at all. For instance, a video surveillance system for traffic control which is based on object identification of cars will require a complete change of the object identification algorithms in order for the system to operate in the vandalism detection domain. Therefore, this kind of systems is not suitable for the multi-domain scenario specified by requirement number 4.

Formal semantic technologies based on ontologies would allow an easy, fast and flexible specification of different operation domains by switching to the appropriate ontology, but its application today is only feasible in environments where cameras have powerful processors and are capable of running complex object identification algorithms. However, design goal number 3 is not compatible with this computing power requirement. This limitation is imposed when operating with a huge number of cameras: embedding powerful processors in all of them would be too expensive, and sending the entire video signal to one control center would require an enormous amount of bandwidth.

This paper presents a system capable of fulfilling all four design goals. This video surveillance system is based on semantic reasoning for performing the image interpretation, so it is possible to easily change the application domain (requirement number 4) by specifying an appropriate ontology. Additionally, semantic reasoning is performed at a high level of abstraction using human concepts (such as “*a car should move along a lane*” or “*there is an alarm if a car is located on a sidewalk*”), thus fulfilling requirement number 2. However, as mentioned, performing this kind of semantic reasoning directly over the video signal would require a lot of computing power, mainly in identifying all objects in the image, and that would be in conflict with requirement number 3. Therefore, the system proposed in this work does not perform object identification directly over the video stream. Instead, the video is preprocessed in a first stage to extract information about moving objects, their size and trajectories. With that information a path model of the scene is created for each camera (*training mode*), and objects are identified on the basis of the parameters of their movement (*operation mode*). Finally, semantic reasoning is performed over this interpretation.

One advantage of this approach is that it facilitates real world deployments of dense networks with many cameras by simplifying the camera-specific calibration and configuration stage. The path model of each scene is built automatically by the system during the learning stage using an unattended route detection algorithm, and the ontology employed is not camera-specific, but domain-specific: concepts defining for instance the normal behaviour of cars (like “cars should be on the road”) are always the same, regardless of the specific road a camera is watching. This means that there is a single ontology for each surveillance domain (traffic control, fire detection, perimeter surveillance, *etc.*) shared by all cameras watching a scene related to that domain. Thanks to this, the only two camera-specific configuration operations required by this approach are: (1) recording camera height and tilt angle at installation time (parameters that will help correct perspective distortion); and (2) selecting the surveillance domain/s to which each camera is applied (that is, selecting the appropriate ontology/ontologies for each camera).

It is worth mentioning that ontology building is a technique on its own, which already has a well-defined set of procedures, tools and best-practices, as reported in the literature [10,12,13,15,25–27]. An ontology built for alarm detection should not be significantly different from ontologies designed for other purposes.

**Figure 1.**
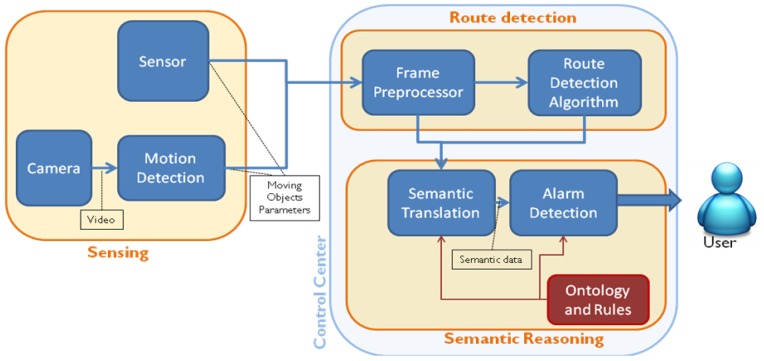
Basic scheme of the proposed surveillance system.

The proposed system is based on a three-stage architecture shown in Figure 1. The first stage of the algorithm is implemented on each sensor camera, and the second and third are located in the system's control center.

These three modules are as follows:

- Sensing: A sensor network including smart surveillance cameras and other sensors (fire and movement detectors, for instance) is connected with the control center. Cameras run motion detection algorithms to transform the video stream into data packets (specifically XML files) that contain information about the different moving objects (speed, position, size, *etc.*).
- Route Detection: Once the XML file with the data of the moving objects is available, trajectories and movement patterns of different objects are processed using an algorithm that builds, for each camera, a route model of the scene (zones of the image where objects usually move) enriched with object sources and sinks (zones of the image where objects usually appear or disappear). Route Detection is implemented with two internal submodules. First, the Frame Preprocessor receives from the camera an XML file with the motion parameters of the objects detected by the camera, separates the integrated data in different frames (a single file can aggregate several frames to optimize communications), corrects the perspective distortion using height and tilt angle values for the source camera by applying a simple Inverse Perspective Mapping [28], and reformats the information in the shape of a raw data matrix. From this data matrix, the Route Detection Algorithm, using a set of routines implemented in Matlab, determines the routes of the scene. Route Detection is performed only when the system is in training mode.
- Semantic reasoning: This stage is only performed in operation mode, when Route Detection stage has finished and the route model of the scene is completed. The aim of this stage is to translate the syntactic parameters of objects, routes, sinks and sources obtained by the cameras and the Route Detection stage into meaningful semantic classes (“car” instead of “object”) and identify any alert situation (a “car is on the sidewalk”) according to the ontology and semantic rules (a formal knowledge model specified by a human ontology engineer). The Semantic Translation translates the syntactic information into formal semantic data (according to Semantic Web standard formats) and populates the ontology with them (using the Jena framework, which handles all the semantic operations done within Java). After the translation, the Alarm Detection submodule processes the ontology (recently populated with new data) with a semantic reasoner to infer new properties about the objects in the image, and specifically, identify if an alarm situation is going on. If it is the case, an appropriate XML Alarm is sent.

In subsequent sections, a thorough description of each piece of the individual modules will be made, in order to understand the system operation.

## 3. Low Level System Design

### 3.1. Sensors

The proposed sensing module consists of a large number of small, cheap and unobtrusive (low resolution) smart visual sensors which are able to communicate wirelessly with a backend control center. Each sensor monitors the motion of objects in a region of about 30 × 30 meters with low resolution (320 × 240 pixels). These low resolution visual sensors make it possible to preserve the privacy of people, not being able to recognize faces (a very important feature for a real Smart City deployment). Even so, video is processed in-camera using motion detection algorithms capable of identifying moving objects and determine their parameters (direction, size, speed, *etc.*). The output of this processing is a stream of light-weight XML (Extensible Markup Language) data packets that contain information about the different moving objects (speed, position, size, *etc.*) in each video frame.

In the prototype implemented inside the HuSIMS project, visual sensors with optimal sensitivity are used. These sensors can operate even in extreme visual conditions, both indoors and outdoors regardless of the lighting and meteorology and have low cost and low power requirements. Emza, one of the project partners, provide their WiseEye visual sensor, a platform capable of covering all the previously mentioned requirements.

It is possible to complement the information from the cameras with other types of sensors such as smoke detectors, humidity and/or accelerometers, to enable a more accurate detection of dangerous situations and detection of alarms not easily identifiable with cameras alone. This data is directly fed to the Semantic Translator, which contains an ontology, a knowledge model representing each surveillance domain considered. For example, a fire detection ontology will probably have to deal with the input of fire sensors, brightness of objects in the cameras (to detect flames and smoke) and speed of people running in the images. All the modules of the system are the same, and even more, all ontologies can be loaded at the same time in the system, it is just necessary to specify which ontologies are applied to each camera. All sensors are connected with the control center using a wireless 4G (WiMAX-Worldwide Interoperability for Microwave Access) [29] network.

### 3.2. Route Detection

The aim of this module is to build a model of the scene watched by each camera using routes and sinks/sources of objects:

- Routes: these are zones (normally strips) of the scene where objects habitually pass through. Each route can be considered as a cluster of similar trajectories.
- Sinks and Sources: these are zones where objects usually appear (sources) or disappear (sinks). It is worth noting that, since cameras only detect objects in motion, a sink could be also a place where an object stops.

Depending on the specific domain of the scene watched, each of these concepts has a different meaning. For instance, when watching a street, routes could be roads and sidewalks, while sinks and sources could be located at the edges of the image and on traffic lights. However, this interpretation is performed in the Semantic Reasoning module.

To perform the route and sink/source detection, the strategy implemented in the system is similar to the one found in [22–24,30–33]. Basically, similar trajectories are clustered together to form routes, with similarity measured using a modified Hausdorff distance composed by two values: the traditionally defined Hausdorff distance, and an average point-by-point angle distance (the average of the angles between the direction of the trajectories at their initial point, at their second point, at their third point, *etc.*). In order for two trajectories to be considered part of the same cluster, the two distance values have to be below two different thresholds. Incorporating the angle measure helps differentiating between two trajectories which have a similar layout but evolve in opposite directions (a problem already reported in [34]).

**Figure 2.**
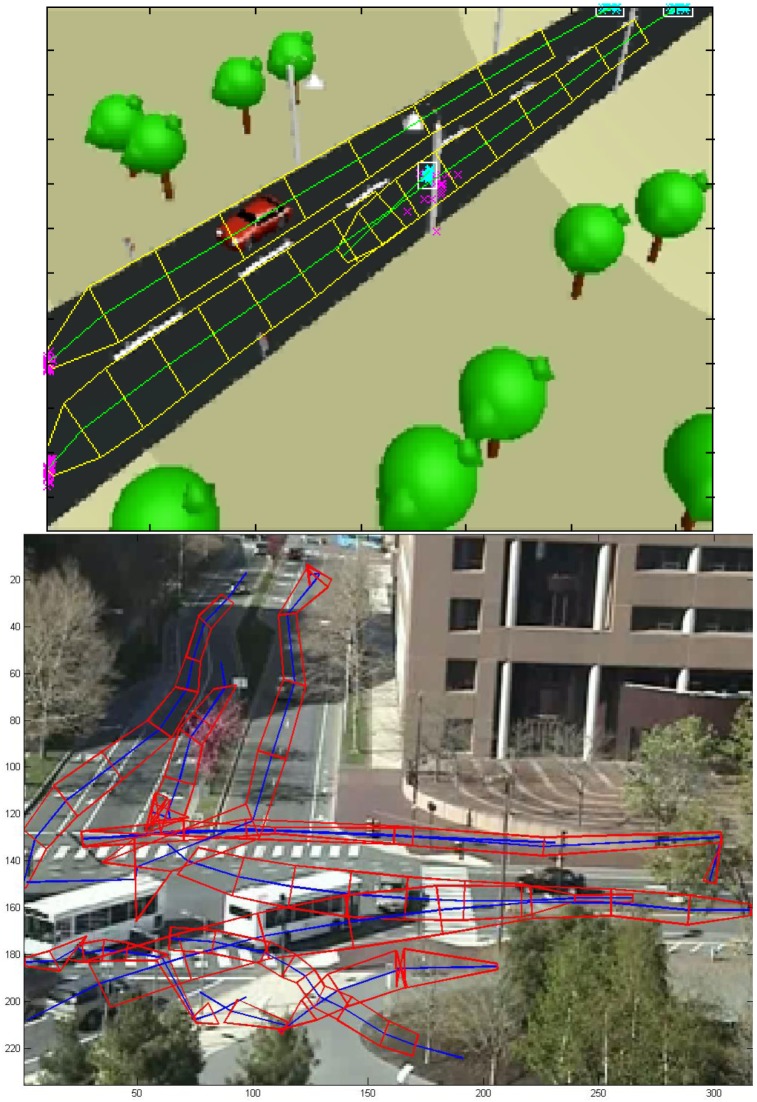
Two example applications (one over a synthetic movie, one over a real movie) of Route Detection. Green and blue lines represent the center line of routes, with yellow and red lines representing the envelopes of the routes. Cyan and magenta “x” points are entry and exit points respectively (a point where an object has appeared or disappeared). White squares are sources (clusters of entry points).

Each route is characterized as a strip: a sequence of center points and two envelopes, as shown in the Figure 2 (the sequence of center points represented by green lines, and the envelopes in yellow lines), marked with a direction (in the figure, the direction of the route is marked with a sharper arrow end). This means that a reversible lane will be represented by two routes, one for each direction. The envelopes are obtained using the width and height values given by the camera.

Additionally, routes present a parameter called “strength” which represents the number of trajectories clustered inside. When a new trajectory matches a route, the points of the resulting route are averaged to find a middle point between the trajectory and the route, but weighting the route with the strength parameter, so well established routes vary less than routes containing only a few trajectories.

Point-by-point comparisons are possible because the trajectories are all resampled with a fixed sample rate. This also has the benefit of reducing the number of points in a trajectory, thus optimizing the amount of computing required for obtaining the Hausdorff distance (which is by definition a costly operation).

The algorithm to build the route model is as follows:

1. Once an object disappears from the screen, the history vector of points he has travelled through is considered its trajectory. The trajectory is then resampled to contain only a (configurable) fixed number of points.
2. Every time a new trajectory is completed:
  - If there is no route in the system, that trajectory is considered a route of strength one.
  - If there is any other route, two distances are computed for each existing route: the Hausdorff distance and an angular distance between the directions of the trajectory and the route at each point.
    - If both distances are under a configurable threshold for only one route, that trajectory is clustered with the route (center points, directions and envelopes averaged with the addition of the new trajectory), and its strength parameter incremented by one.
    - If both distances are under a configurable threshold for several routes, that trajectory is clustered together with the nearest route in the same way described in the previous point.
    - If no distance is under the thresholds, then a new route of strength one is created with only the trajectory under consideration.
3. The initial and final points of the trajectory vector are added to a source and sink matrix respectively. The points in those matrixes are clustered using the Gustafson-Kessel (GK) clustering algorithm [35] to obtain groups of sinks and sources. The zones of the scene defined by those clusters are considered sinks and sources. Figure 2 represents all the sink points as magenta crosses, all the source points as cyan crosses, and the source clusters produced by the GK algorithm as white squares.

Additionally, routes also contain a set of parameters about the objects trajectories that are clustered inside: average size, average maximum speed and average mean speed, in the case of traffic control, but others could be included for other domains. This will help the Semantic Translator to identify the kind of objects which use that route, and therefore assign a proper meaning to it. For instance, in a traffic control scenario, routes with average size and speed higher than the mean average size and speed of all the routes, can be considered candidates (specified in the ontology) to be roads, and the others, sidewalks.

### 3.3. Semantic Processing

The Semantic Reasoning stage is composed of two substages, Semantic Translation and Alarm Detection. It is worth noting that at this stage the real time data of each object is considered and analysed: alarm detection is not performed using the trajectory of each object (which is only available after the object disappears from the scene and used to compute the route model).

- Semantic Translation: Data obtained by each sensor camera is processed according to the route model built by the Route Detector and the semantic knowledge model (comprised by an ontology and a set of rules), with the aim of transforming the syntactic data about moving objects into semantic data. For that, sinks, sources, routes and objects are assigned to one of the classes in the ontology depending on its parameters. For instance, a specific route can be labelled as a sidewalk or a road depending on the average and maximum size and speed of objects that usually follow it; a specific object can be labelled as a car or a pedestrian depending on its speed and size; a sink can be labelled as a door if pedestrians normally disappear through it.
- Semantic Alarm Detection: Once the image and the route model have been translated into the semantic domain, the ontology and rules are applied to check if the behaviour of the object is normal or there is an emergency. For instance, a semantic rule could specify that “cars do not move over sidewalks”, so an object labelled as “car” on a route labelled as “sidewalk” will trigger an alarm.

The semantic analyzer module employs a semantic knowledge model which represents the considered surveillance domain. This knowledge model comprises an ontology and a set of semantic rules, and is built by a human ontology engineer.

- The Ontology is an abstract representation of all the different actors involved in the considered domain, as classes (abstract groups of similar actors) and individuals (specific instances of a class) together with their properties and the relationships among them. It could be viewed as a kind of extended UML (Unified Modeling Language) class and instance diagram which allows for a more complex specification of properties and relationships. For instance, a traffic ontology could specify a class “vehicle” with the properties “speed” and “located_in”, and the subclasses “car” and “truck”, and the class “lane”. The individual “individual_1” could be a car with a *speed* of 40 Km/h and *located_in* “lane_1” and “individual_2”, a truck with a *speed* of 30 Km/h *located_in* “lane_2”. It is worth mentioning that perspective distortion in speed and size measures is corrected by using the tilt angle and height measures of a camera, which are configured by the technician at installation time and stored in a database at the system's control center.
- The set of semantic rules are a formal specification of conditions and logic operations to be performed over the ontology. For instance, a rule can specify that whenever the position of a member of the “moving object” class is inside a member of the “route” class, the property *located_in* of the former is set pointing to the latter; another rule could specify that whenever an object of the class “car” is located over an object of the class “sidewalk”, the status of the “car” object should be changed to “alarm”.

**Figure 3.**
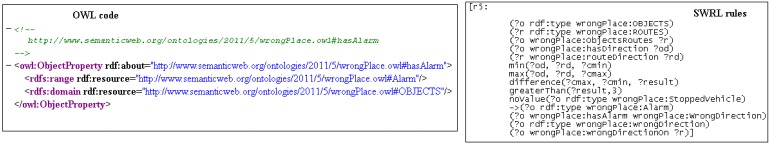
OWL and SWRL code.

The prototype implemented uses Semantic Web standards for the specification of these two elements, using the JENA framework as a provider of all the required semantic operations within Java. Specifically, the ontology is defined using OWL (Web Ontology Language); SWRL (Semantic Web Rule Language) is used for the specification of the semantic rules (Figure 3). The ontology governs how the moving objects, routes, sinks and sources are understood. For instance, in a traffic management ontology used for a camera watching a segment of a highway, moving objects will be cars and trucks; routes will be highway lanes; sinks and sources will most probably be the edges of the highway segment. But in a vandalism control ontology used for a camera watching a public garden, moving objects will be people, and routes will be common pathways.

When the system is operating, cameras send data to the semantic translator through the frame preprocessor. The semantic translator makes an interpretation of the frame received and updates the ontology adding the appropriate individuals to their appropriate classes. This is done with the help of the semantic rules, which specify the conditions that have to be met by an object to be considered a member of a specific class.

A semantic reasoner (a program designed to infer conclusions from the specification of classes and individuals within an ontology) is in charge of reasoning over the ontology and of applying the semantic rules. Every time new data is available, the reasoner processes it, updating the knowledge model of the scene accordingly. Pellet has been chosen as the reasoner in the implementation.

**Figure 4.**
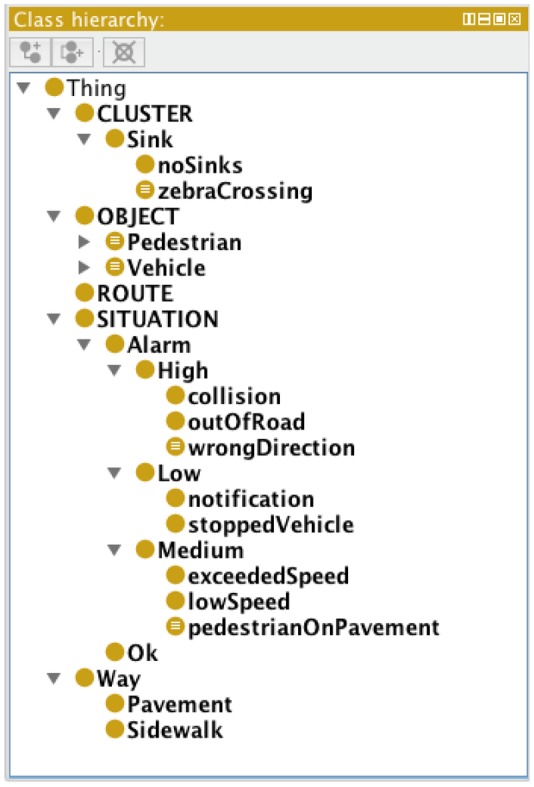
Hierarchical structure of ontology.

**Figure 5.**
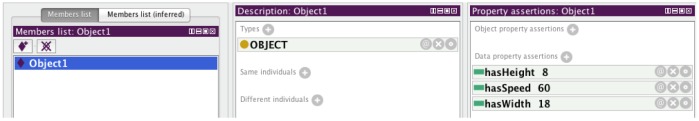
Unclassified individual.

**Figure 6.**
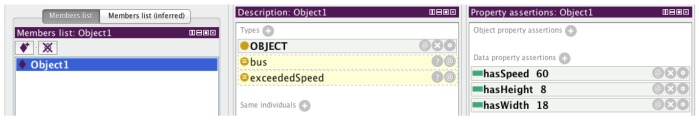
Classified individual.

Figure 4 shows the hierarchical class structure of an example traffic domain ontology designed to test the system. Figure 5 shows the description of an individual with its own characteristics prior to its classification by Pellet. Figure 6 shows how this object has been classified according to the semantic rules specified, with two new classes to which the object belongs to appearing in its description. Firstly, it is classified as “bus” within the “vehicle” category and, in addition, it is classified as “exceededSpeed”, within the “Alarm” class, as the appropriate conditions are met.

It is worth noting that this case presents an “absolute” definition of the conditions for “exceededSpeed” alarm that takes advantage of the parameter normalization made at the frame preprocessor. In this case, the ontology should be modified depending on the specific conditions of the road where the camera is located (varying for instance between a highway and a downtown street). However, a “relative” definition of the alarm is also possible, by launching the alarm for instance when an object surpasses by some threshold the average maximum speed of past objects in the same route.

One advantage of the proposed system is that it is capable of embracing different domains by simply designing an appropriate ontology and an appropriate set of rules. This makes the system extremely flexible and configurable according to the specific domain in which it is operating. For instance, inside a Smart Building, ontologies for fire detection and crowd control could be used, while for a Smart City traffic control and vandalism detection would be more suitable. In both cases the system employed will be the same, with the only exception of the ontologies and the rules.

Additionally, specific manual configuration of each camera is not required. As the route detection process is camera specific, the scene model for each camera is built dynamically in the control center. Even more, thanks to the perspective distortion correction done at the Frame Preprocessor component, the numeric size and speed values are specified generically in the ontology, and then applicable across all cameras.

## 4. Uses Cases

The system has been tested with real video data in order to validate the approach. This section presents three use cases where the operation of the system is shown. In all of them, the video data set was processed using the Wise Eye software, so the results are exactly the same as if a Wise Eye was physically present in the place of original camera.

### 4.1. Pedestrian Crossing the Street Inappropriately

In the first two use cases the benchmark data employed is the MIT Traffic Surveillance Benchmark (available at [36]), and the route model extracted for both of them has been shown in Figure 2.

**Figure 7.**
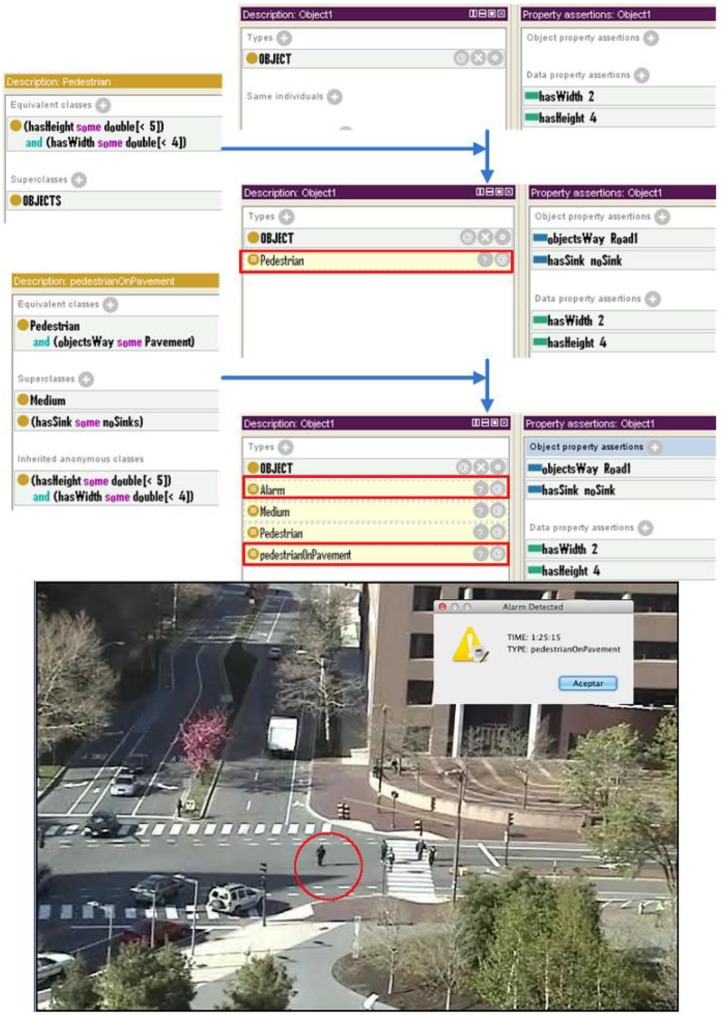
Example of semantic reasoning for pedestrian use case.

This first scenario (Figure 7) may happen in any urban area of any city. A camera controls a scene comprised by several roads and sidewalks controlled by traffic lights, including a bicycle lane, and the vehicles and people moving over them. At a particular time a pedestrian crosses the road improperly without using the crosswalk.

In this case the ontology classifies a new individual (the pedestrian crossing improperly) according to the values of the properties *hasHeight* and *hasWidth*. In this case the individual has values *2* for *width* and *4* for *height*, so it is assigned by the reasoner to the *Pedestrian* class.

At the same time, two additional properties of the individual have been automatically filled by the reasoner using the semantic ruleset: *objectWay* and *hasSink*. The property *objectWay* specifies the road type on which it is located. In this case, the *Pedestrian* has this parameter set to *Pavement*. Previously, the system has inferred that the route *Pavement* is a road because of the average speed and size of objects that have crossed through it. The *hasSink* property, also automatically fulfilled by the reasoner according to the semantic ruleset, indicates that the pedestrian is outside a *zebraCrossing*.

Therefore, the semantic reasoner infers that an alarm of the type *pedestrianOnPavement* is happening.

In order to avoid false alert due to spurious readings, the system checks that this alarm is happening for a minimum period of time, and then the system launches an alarm towards the control center indicating that a pedestrian is crossing the road improperly. One of the advantages of the semantic approach implemented in the proposed system is that the alarm includes detailed data about the event that caused it. In this case, the alarm *pedestrianOnPavement* might not deserve the attention of a human operator (because this kind of alarms happen often and are not extremely dangerous), so the system could automatically decide that the alarm should not be progressed to the control console in this case, according to a behaviour that can also be encoded directly in the ontology. This feature is extremely convenient in Smart Environments, where automatic actuators of contingency systems could be automatically triggered by the surveillance system (for instance, fire extinguishers in a Smart Building), therefore allowing the specification of semantic conditions for automatic responses to each kind of alarm.

### 4.2. Vehicle in the Wrong Direction

**Figure 8.**
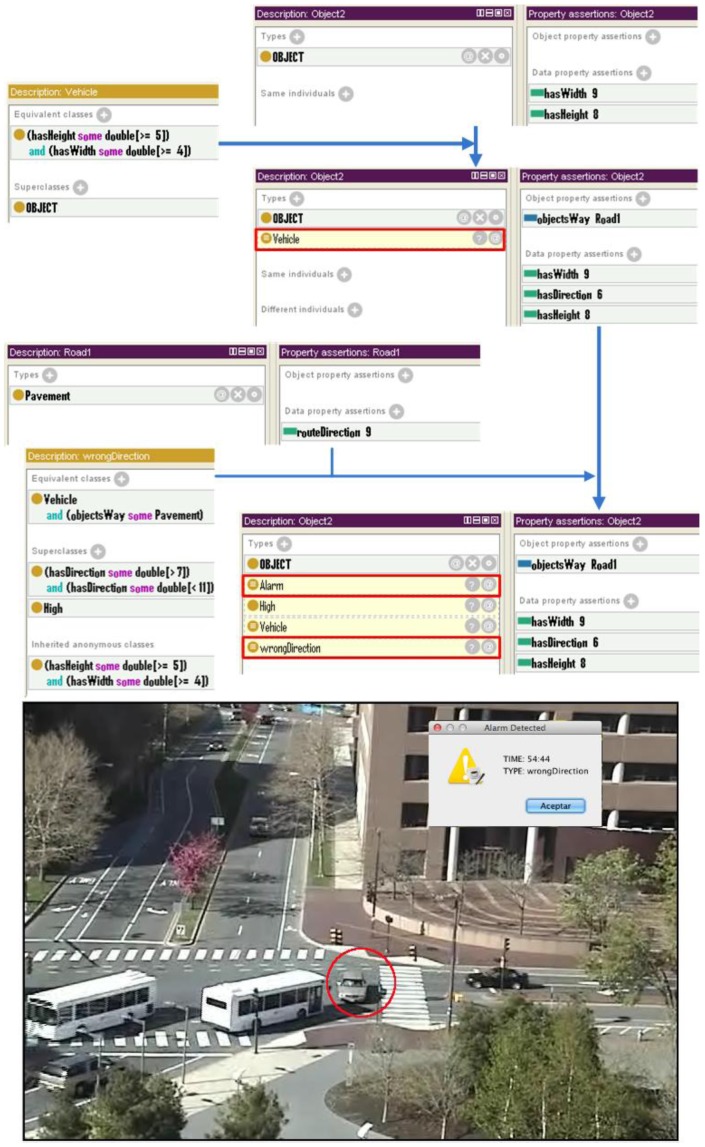
Example of semantic reasoning for vehicle use case.

In this scenario a car circulating in the wrong direction is causing a risk to the rest of the vehicles (Figure 8). Like in the previous case, the ontology classifies the individual as a member of the *Vehicle* class due to the values of the properties *hasWidth* and *hasHeight* (9 and 8 respectively, and therefore within the predefined ranges of the *Vehicle* class).

In this case the reasoner, according to the rules, has assigned this *Vehicle* the parameter *Pavement* for its property *objectWay*, meaning that it is located in the *Pavement*. However, this *Pavement* (a member of the class *Way*) presents a property *routeDirection* that determines the direction of objects that circulate through it. To verify that the *Vehicle* is in the right direction for the *Pavement* in which it is located, the semantic reasoner analyzes the value of its property *hasDirection*. As the direction of the *Vehicle* does not match the appropriate one of the *Pavemenent* in which it is located, the system throws an alarm of the type *wrongDirection*.

### 4.3. Pedestrian in the Railway

In the previous two use cases, the system works in a traffic control scenario, but the system is easily ported to other environments, maintaining all the software and hardware modules and just changing the ontology employed in the semantic reasoning and translation.

**Figure 9.**
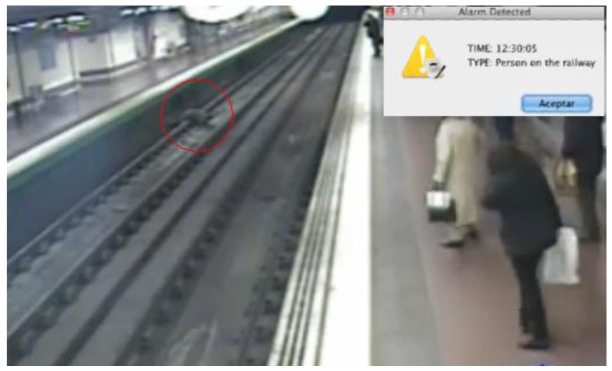
Subway Alarm.

In this scenario the system is employed as a security means to detect when a person falls to the subway tracks in a station (Figure 9). In that situation, the ontology classifies the individual as a *Pedestrian* who is walking in the platform. When the pedestrian falls and is located on the tracks, the system raises an alarm.

It is worth noting that if an object falls to the tracks, when it lands it is no longer detected by the camera as an object because it is no longer in motion. However, the system understands that one object that was moving has disappeared in a place that is not a sink zone, so an alarm is raised too.

A surveillance engine based on statistical analysis could also detect that there has been an alarm because an object has been detected moving in a zone and with a direction that do not normally happen. However, in that case, it is impossible to automatically differentiate between a person falling to the tracks and one person climbing over a bench, for instance. That means that a human operator has to check the video signal to identify the alarm conditions and, in this case, call the subway driver to warn him about the fallen person. This process may take some time, time that in critical situations like this may not be enough.

On the contrary, the semantic system proposed in this work presents the advantage of being able to discriminate the kind of alarm that is happening, and in this case, the system might be configured (specifying a specific behaviour in the ontology) to automatically send the alarms of the type “Person in the tracks” to the subway drivers, or even more, to activate the emergency brakes of the train entering the station. The literature [4,6–8] presents cases of special purpose systems based on semantics that are able to offer this alarm response automation feature as well. However, in those cases the semantics of the surveillance domain are hardcoded in the system, so it is only applicable in that specific domain. On the contrary, in the system presented along this work the semantics are separated from the system implementation, so changing the ontology is enough to have exactly the same system operating across different domains.

## 5. Evaluation Results

In order to characterize the behaviour of the proposed system, it is important to study both the resource consumption and the accuracy of the system when identifying the alarms. According to the system's architecture, comprised by the route detector and the semantic processing module, this analysis has been performed independently to identify the different features of each component.

The behaviour of the route detector has been studied both in terms of resource consumption and accuracy in route identification. For that, four different videos have been analyzed by the route detector, recording the processing time consumed and manually comparing the paths identified with the real paths to be detected, so as to calculate the % of correctly identified paths and the % of wrong paths identified (for an ideal operation, these figures would be 100% and 0% respectively). The computer employed for the trials was an Intel Pentium 4 3.00 GHz with 2 GB RAM. Table 1 presents the results of the analysis.

The number of objects appearing in each video and the number of points per trajectory considered in each execution have also been included. When examining the figures for each video it is easy to realize that the number of different routes in the image impacts the processing time to the point that the most complex videos apparently cannot be processed in real time. However, it has to be remembered that route detection is only executed at learning time, so the algorithm does not need to operate in real time.

For instance, the most complex scene analyzed consumes a processing time of 1,696 seconds; considering a re-learning rate of once a day (a quite high rate for a Smart City), the number of cameras that a single machine like the old one employed for the tests can support is more than 50.

Regarding accuracy, the numbers for simple scenes, like the ones expected for highways with well defined lanes, are very good, easily identifying all the lanes with no errors when running the algorithms with 10 point trajectories or more. In more complex scenes, the rates are still good for big objects (cars) and not so good for small objects (people). This high false-positive rate happens because a very limited resolution is employed for analyzing videos originally recorded with much higher resolution. Therefore, the algorithm has a lot of potential for improvement when placing the cameras nearer to the floor (or using higher resolutions and/or more accurate—but heavier—motion detection algorithms), but results under these conditions are satisfactory for a lot of applications in Smart Cities.

Regarding semantic processing (which includes the semantic translation and semantic reasoning subprocesses), a synthetic benchmark has been developed to identify the influence of the number of routes and objects in the scene. At this point of the system, the data considered is no longer an image, but only individuals inside an ontology, so there is no dependency with visual/spatial magnitudes like geometry or resolution of the scene, and therefore the results of the benchmark are directly portable to real operation conditions. A conclusion of this is that the number of points per trajectory considered has little to no impact in the semantic analysis stage.

**Figure 10.**
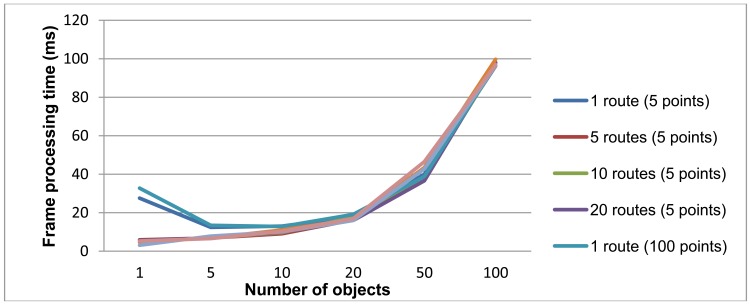
Frame processing time against number of objects and routes in the image and points-per-trajectory parameter.

For each test, 50 frames were generated with random locations for the appropriate number of objects and routes, and then processed with the semantic engine in the same machine used for the route detector evaluation, an Intel Pentium 4 3.00 GHz with 2 Gb RAM. Figure 10 presents the average processing time for each frame.

The inference time per frame depends mainly on the number of objects. Variations in the number of routes or the number of points per trajectory give very similar results. The results are quite satisfactory, since a frame with less than 20 objects is processed in around 10 ms, and an extremely busy scenario with 100 objects is processed in less than 100 ms.

Tests about alarm identification accuracy and false alarm rates are of little value when applied to the semantic stage of the system. Due to its semantic nature (that is, the conditions for raising an alarm are defined in terms of the meaning of the objects and their behaviour), if the scene is properly characterized by the route detector and the ontology considers the appropriate conditions, 100% identification rates and 0% false alarms are usual. Therefore, the accuracy of the system depends directly on the route detection rates (which in turn depend heavily on the quality of the motion detection algorithms).

## 6. Discussion

In the last years autonomous surveillance systems have been a hot topic. The need for large area systems covering big spaces render human based systems obsolete. The available solutions documented in the literature, such as [37], employ two different strategies for alarm identification: either statistical analysis (they mathematically characterize the normal variations of the image parameters and identify situations outside that characterization) or “semantic” analysis (the meaning of the objects in the image are employed to understand when an alarm situation is developing).

In the literature, the word “semantic” [22,38–41] is sometimes used to specify that alarm detection is not done only statistically (like in [42]), but is tightly bound to the domain of the scene watched, so detection of alarms is made out of the meaning of the things that happen in the image, which is hardcoded inside the alarm detection algorithms. For instance, a system for the detection of speed excess in a highway could perform object identification to detect cars and scan their speed, but that algorithm cannot be modified to detect fire alarms or suspicious people running in a building. This work goes a step further than this approach, because it is based on the formal representation of the meaning of the image, using Semantic Web technologies, that allow fast and easy ontology design. Therefore, just modifying the ontology the owner can switch the alarm conditions, the parameters employed and even the entire surveillance domain.

The other use of the word “semantic” in the literature is in papers that actually employ formal Semantic Web technologies. However, these approaches only use semantic technologies for characterizing information. Zhang *et al.* [43,44] and Marraud *et al.* [45] in their studies, use semantics to allow advanced queries based on video features to databases; François *et al.* [46] propose a new language for the representation of events in a video to facilitate searches, and Poppe *et al.* [47] report about a system to unify the video surveillance semantic information. That means that they employ some other approach to identify objects and alarms, and then use semantic technologies only to represent that information. This approach is different from those because it is fully semantic: the information is first translated to the formal semantic domain, and then reasoning, object identification and alarm detection is performed using pure semantic reasoning.

Against statistical analysis, the solution proposed in this paper presents the advantage that it is able to send enriched information about the identified alarms, and it is much more controllable and predictable because an expert is always able to inspect the ontology and semantically specify or modify alarm conditions. Smart Cities are able to seize this advantage, because this rich information can be used to drive automatic responses to alarms.

## 7. Conclusions

In traditional video surveillance systems a human operator is required for interpretation of the scene and to perform the necessary actions when an alarm is verified. This might not be a big problem when the operator is watching a limited area with a limited number of cameras. However, as the number of cameras to watch grows, several operators might be required, increasing the complexity and cost of the system. Automated surveillance systems have appeared to solve this problem, packing algorithms for the autonomous interpretation of the scene and the identification of alarm conditions.

However, in Smart Spaces, dense and flexible camera networks are usually required, with many cameras to cover wide areas in Smart Cities or Smart Buildings. Most of the documented autonomous systems are not suitable for these environments, since they require too many resources or are based on hardcoded approaches.

The system implemented along this work, which has been carried out inside the Eureka Celtic HuSIMS project, overcomes these difficulties by working with cheap, low resolution cameras and following a light-weight, data-based approach for behaviour identification. The result is that a lot of cheap cameras can be deployed without requiring a huge bandwidth in the system or processing power in the cameras. In addition, the semantic approach followed allows rich alarm detection, adding a lot of meaningful information to each emergency triggered, and therefore opening the door to the implementation of autonomous responses using the actuators of the Smart Space under surveillance.

Being based on the semantic reasoning over a formal knowledge model implemented by a human operator, the system is suitable for its deployment in a wide range of different environments, only by switching to the appropriate ontology and ruleset. While some systems based on statistical analysis which are not domain-specific may operate in different domains without any kind of adaptation, they present several disadvantages with respect to the solution presented here: their behaviour is difficult to predict (since they are based on mathematical analysis of the video parameters) and is not related to the semantics of the scene watched, they are unable to discriminate among different types of alarms and cannot give rich information about them. Therefore, they are in general unsuitable for its deployment within Smart Spaces.

In short, the semantic-based approach for the detection of alarms in video surveillance systems presented in this paper offers a number of important advantages that are not available in other solutions in the current state of the art.

## Figures and Tables

**Table 1.** Performance results for the route detector. N is the number of points per trajectory.

**Video Description**	**N**	**Duration (s)**	**Processing Time (s)**	**Number of Objects**	**Number of Paths**	**Number of Paths Identified**	**Number of wrong paths identified**	**% identified paths**	**% wrong paths**
A synthetic video of a highway with two lanes, one direction per lane.	5	600	318s	359	2	2	2	100%	100%
	10	600	283s	359	2	2	0	100%	0%
	20	600	402s	359	2	2	0	100%	0%
A synthetic video of a highway with four lanes, two lanes for each direction.	5	480	26s	58	4	3	1	75%	25%
	10	480	52s	58	4	4	0	100%	0%
	20	480	123s	58	4	4	0	100%	0%
A real video of a complex intersection, involving three roads for cars with two directions, and several sidewalks.	5	180	118	191	6 (cars) 7 (people)	6 (cars) 2 (people)	1 (cars) 1 (people)	100% (cars) 28% (people)	16% (cars) 14% (people)
	10	180	284	191	6 (cars) 7 (people)	6 (cars) 2 (people)	1 (cars) 1 (people)	100% (cars) 28% (people)	16% (cars) 14% (people)
	20	180	1696	191	6 (cars) 7 (people)	5 (cars) 2 (people)	2 (cars) 1 (people)	86% (cars) 28% (people)	33% (cars) 14% (people)
Part of the MIT video benchmark showing a complex intersection with roads and sidewalks.	5	202	196	145	6 (cars) 6 (people)	3 (cars) 1 (people)	3 (cars) 1 (people)	50% (cars) 16% (people)	50% (cars) 16% (people)
	10	202	831	145	6 (cars) 6 (people)	4 (cars) 1 (people)	2 (cars) 1 (people)	66% (cars) 16% (people)	33% (cars) 16% (people)
